# Coronavirus 2′-*O*-methyltransferase: A promising therapeutic target

**DOI:** 10.1016/j.virusres.2023.199211

**Published:** 2023-09-04

**Authors:** Craig Schindewolf, Vineet D. Menachery

**Affiliations:** a Center for Immunity and Immunotherapies, Seattle Children's Research Institute, Seattle, WA, USA; b Department of Microbiology and Immunology, University of Texas Medical Branch, Galveston, TX, USA; c Institute for Human Infections and Immunity, University of Texas Medical Branch, Galveston, TX, USA; d World Reference Center for Emerging Viruses and Arboviruses, University of Texas Medical Branch, Galveston, TX, USA

**Keywords:** Coronavirus, NSP16, IFIT1, IFIT, IFIT3, Antiviral, 2′O methyl-transferase, Capping

## Abstract

Coronaviruses (CoVs) have been the source of multiple epidemics and a global pandemic since the start of century, and there is an urgent need to understand CoV biology and develop better therapeutics. Here, we review the role of NSP16 in CoV replication, specifically its importance to 2′-*O*-methylation and CoV RNA capping. We describe the attenuation phenotypes of NSP16-mutant CoVs, the roles of MDA5 and IFITs in sensing and antagonizing viral RNA lacking 2′O methylation, and the dependence on 2′-*O*-methylation in other virus families. We also detail the growing body of research into targeting 2′-*O*-methylation for therapeutics or as a platform for live attenuated vaccines. Beyond its role in RNA capping, NSP16 may have yet uncharacterized importance to CoV replication, highlighting the need for continued studies into NSP16 functions. Understanding the full contribution of NSP16 to the replicative fitness of CoVs will better inform the development of treatments against future CoV outbreaks.

## Highlights

• Coronavirus NSP16 plays a critical role in viral RNA capping and evasion of innate immunity.
• Coronavirus mutants lacking NSP16 activity are attenuated both *in vitro* and *in vivo*.
• Attenuation of NSP16 mutants driven by activity of IFIT family members targeting viral RNA lacking a 2′O methylation on its cap.
• Targeting NSP16 activity offers a broad therapeutic approach for treatment of current and future coronavirus outbreaks.

## 1 Introduction

Prior to the 21st century, coronaviruses (CoVs) were considered pathogens of minor concern to human health, known to cause cold-like symptoms and infrequently associated with more severe respiratory disease (Riski and Hovi, 1980). The current perception of CoVs is now much different. In 2019, severe acute respiratory syndrome CoV 2 (SARS-CoV-2), the causative agent of COVID-19, emerged in Wuhan, China under conditions that still remain unclear (Wang et al., 2020). Initially termed the Novel Coronavirus (nCoV)−2019, the virus bore strong genetic resemblance to circulating viruses of the *Sarbecovirus* subgenus, or SARS-related CoVs, found in bats (Zhou et al., 2020). The prototypical SARS-CoV had previously emerged in 2002 in China (Low and McGeer, 2003). While more phylogenetically distant from the SARS-related CoVs, Middle East respiratory syndrome (MERS)-CoV is also capable of causing severe respiratory distress (Lamers and Haagmans, 2022), and continues to circulate at low frequencies in the Middle East (World Health Organization, 2022). Thus, the emergence of three highly pathogenic human CoVs since the start of the 21st century, combined with the presence of circulating CoVs in natural reservoirs, such as bats, underscores the persistent threat of future CoV emergence, the need to better understand CoV biology, and the potential benefits of developing better treatment strategies.

## 2 CoV replication and the nonstructural proteins

CoVs are positive-sense, non-segmented RNA viruses, which possess large RNA genomes, up to 31.7 kgbases (kb) (Woo et al., 2010). The viruses belong to the order *Nidovirales* (nidoviruses), and are so named because of the 3′ co-terminal, or nested (Latin: “*nido*”), set of sub-genomic RNA species that are generated during transcription of the viral genome. In addition to CoVs, nidoviruses include the newly distinguished *Tobaniviridae* family (which include the toroviruses), the genetically smaller viruses of the *Arteriviridae* family (arteriviruses), invertebrate viruses of the *Roniviridae* and *Mesoniviridae* families, and novel families recently delineated by the International Committee on Taxonomy of Viruses (https://ictv.global/taxonomy) (Gulyaeva and Gorbalenya, 2021; Snijder et al., 2016).

As positive-sense RNA viruses, nidoviruses, including CoVs, initiate translation of the viral genome upon release into the cytoplasm (V'kovski et al., 2021). Importantly, a large ORF at the 5′ end of the genome, comprising about two-thirds of the genome, ORF1ab, encodes a replication-transcription complex (RTC) which comprises, in CoVs, sixteen distinct nonstructural proteins (NSPs). The RTC localizes to virus-induced, endoplasmic reticulum (ER)-derived double membrane vesicles (DMVs), which serve as virus replication factories; the DMVs also likely shield viral RNA from host pattern recognition receptors (PRRs) that detect viral RNA (Horova et al., 2021; Klein et al., 2020; Snijder et al., 2020; V'kovski et al., 2021). The NSPs perform such essential functions as replicating and transcribing the genome, capping the viral RNA, and proteolytically processing the ORF1ab polyprotein into the individual NSPs (Romano et al., 2020).

Nidoviruses possess many shared features of replication, including a polycistronic genome structure, the aforementioned ORF1ab RTC polyprotein that consists of two slightly overlapping ORFs (ORF1a and ORF1b), an encoded ribosomal frameshift mechanism that permits the extension of the second of these two ORFs from the first, a conserved nucleotidyltransferase domain adjacent to the RNA-dependent RNA polymerase (RdRp), and a conserved helicase-containing domain (Lehmann et al., 2015). Notably, CoVs possess NSP functions that are lacking in other nidoviruses (Snijder et al., 2016). Compared to the smaller genomes of arteriviruses, CoV genomes encode additional functional components including an RNA exonuclease (ExoN, NSP14), important for proofreading during replication (Denison et al., 2011), a guanine-N7 methyltransferase (N7-MTase, encoded on a separate domain of NSP14), and a 2′-*O*-MTase (NSP16), the latter two important in viral RNA capping (Lauber et al., 2013).

## 3 CoV RNA capping

**Fig. 1:**
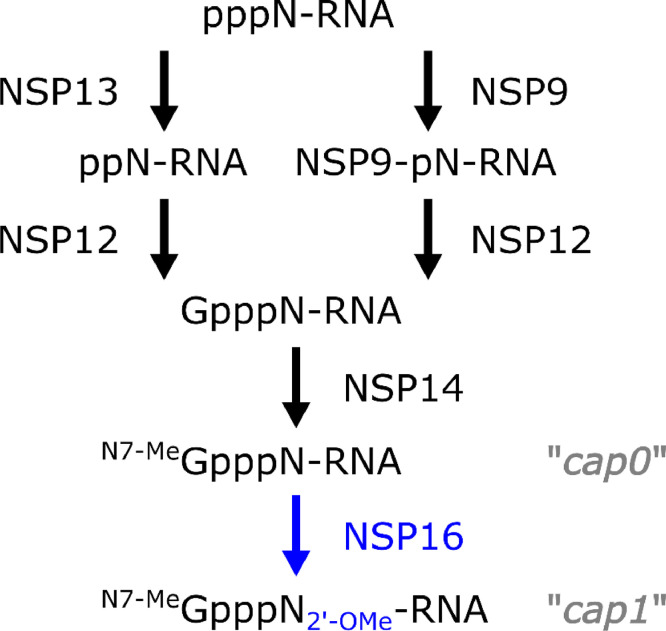
**RNA Capping Model in CoVs.** Newly synthesized viral RNA contains a 5′ triphosphate, which may be cleaved by NSP13 (Ivanov and Ziebuhr, 2004) followed by NSP12-mediated guanosine cap transfer (Walker et al., 2021). Alternatively, NSP9 may form an intermediate with the viral RNA itself prior to guanosine cap transfer (Park et al., 2022). Regardless, NSP14 methylates the N7 of the guanine base of the cap, resulting in cap0 RNA (Chen et al., 2009). Finally, NSP16 methylates the 2′-*O* of the ribose of the first transcribed nucleotide, resulting in cap1 RNA (Decroly et al., 2008).

The CoV 2′-*O*-MTase, NSP16, catalyzes the transfer of a methyl group to the viral RNA cap (Benoni et al., 2021; Viswanathan et al., 2020). This modification prevents recognition by host PRRs, especially effectors in the interferon-induced protein with tetratricopeptide repeats (IFIT) family (see below) (Daffis et al., 2010; Züst et al., 2011). Not surprisingly, numerous viral families have developed strategies to utilize 2′-*O*-MTase activity to evade host immunity, including using host capping machinery (Bergant et al., 2022; Ringeard et al., 2019; Russ et al., 2022), cap snatching (Reguera et al., 2016), or encoding their own 2′-*O*-MTases (Li et al., 2005; Schnierle et al., 1992; Stewart and Roy, 2015; Tay and Vasudevan, 2018; Valle et al., 2021; Zhang et al., 2014). For CoVs, virally encoded 2′-*O*-MTase function is conserved in nidoviruses in general, with the exception of the arteriviruses (Ferron et al., 2020). CoV NSP16 prefers RNA methylated at the N7 position of the guanosine cap, also known as cap0 structure, as substrate (Benoni et al., 2021; Bouvet et al., 2010). This suggests that N7 methylation of the guanosine cap by NSP14 precedes 2′-*O*-methylation of the first transcribed nucleotide by NSP16, resulting in cap1 RNA structure (Bouvet et al., 2010). The nature of the guanosine transfer, which precedes guanine-N7 methylation, was recently shown to be facilitated by the highly conserved NiRAN (nidovirus RNA-dependent RNA polymerase-associated nucleotidyltransferase) domain of NSP12 (Park et al., 2022; Walker et al., 2021). In this context, NSP13, a helicase-containing protein which possesses RNA 5′-triphosphatase activity (Ivanov and Ziebuhr, 2004), may hydrolyze the 5′-terminal γ-phosphate of the nascent viral RNA, a prerequisite for guanosine transferase activity. NSP9 may also serve as an intermediate substrate for viral RNA attachment immediately before guanosine cap transfer (Park et al., 2022) (Fig. 1). In summary, the 2′-*O*-MTase function of NSP16 is the last step in RNA capping and depends on the prior steps of CoV RNA capping mediated by other NSPs. Conversely, CoVs with the NSP16-coding region deleted or interrupted with a premature STOP codon appear non-viable (Almazán et al., 2006; Schindewolf et al., 2023), suggesting, perhaps, that CoV replication and the RTC may depend in some uncharacterized manner on the presence of NSP16.

## 4 Conservation of NSP16

NSP16 is among the best-conserved NSPs within the CoV family (Abbasian et al., 2023; Li et al., 2023a), highlighting both its importance to CoVs as well as its potential druggability. Among the seven known CoVs that infect humans (SARS-CoV, SARS-CoV-2, MERS-CoV, human [H] CoV-OC43, HCoV-229E, HCoV-HKU1, and HCoV-NL63), conservation of NSP16 amino acid sequences is about 53% across the entire CoV family (Li et al., 2023a). However, a tetrad of four amino acids—lysine, aspartate, lysine, and glutamate (at amino acid positions 46, 130, 170, and 203 in SARS-CoV-2)—is conserved across these seven human-infecting CoVs and constitutes an invariant catalytic core of the 2′-*O*-MTase, requiring each residue for activity (Decroly et al., 2008). Indeed, this KDKE amino acid signature is a hallmark of viral 2′-*O*-MTases in general, present in flaviviruses (Egloff et al., 2002), filoviruses (Martin et al., 2018), paramyxoviruses (Li et al., 2023b), poxviruses (Zhang et al., 2016), rhabdoviruses, (Rahmeh et al., 2009), pneumoviruses (Guan et al., 2003), and reoviruses (Sutton et al., 2007), and appears both highly conserved and highly sensitive to functional perturbation (Ray et al., 2006; Zhou et al., 2007).

In addition to the KDKE motif, NSP16 MTase function depends on binding NSP10. Providing structural stability for both NSP16 and NSP14 (Krafcikova et al., 2020), NSP10 is required for NSP16 capacity to bind both RNA substrate and the methyl donor *S*-adenosyl-l-methionine (SAM) (Chen et al., 2011). Like NSP16, NSP10 is also well-conserved among CoVs (Abbasian et al., 2023; Li et al., 2023a). In the absence of NSP10, NSP16 MTase function is completely abrogated in biochemical studies (Bouvet et al., 2010). Targeting small peptides to the NSP10-NSP16 interface to prevent binding demonstrated that such peptides reduce NSP16 MTase activity and CoV replication *in vitro.* In addition, targeting the NSP10-NSP16 interface even protected mice from a uniformly lethal dose of 5 × 10^5^ plaque-forming units (PFU) MHV-A59 administered intrahepatically, compared to a control peptide with scrambled sequence (Wang et al., 2015). Together, these results argue that both the KDKE motif as well as the interaction with NSP10 are necessary for CoV MTase activity.

## 5 Coronavirus NSP16 mutants

In CoVs, the functional consequences of 2′-*O*-methylation on viral replication and pathogenesis are well established, including in mouse hepatitis virus (MHV) (Daffis et al., 2010; Züst et al., 2011), HCoV-229E (Züst et al., 2011), SARS-CoV (Menachery et al., 2014b), MERS-CoV (Menachery et al., 2017), and SARS-CoV-2 (Bergant et al., 2022; Russ et al., 2022; Schindewolf et al., 2023; Ye et al., 2022). These studies all utilized mutations in the conserved KDKE catalytic tetrad necessary for NSP16 MTase function (Jiang et al., 2020; Züst et al., 2011). Confirming biochemical studies (Bouvet et al., 2010; Decroly et al., 2008), infection with CoV 2′-*O*-MTase mutants provided indirect evidence of a lack of 2′-*O*-methylation on viral RNA (Züst et al., 2011). Mutating the aspartate (D) or either lysine (K) (with or without additional mutations in the tetrad) resulted in attenuated CoVs in cells that produced type I interferon (IFN-I) or were administered IFN-I exogenously (Menachery et al., 2017, 2014b; Russ et al., 2022; Schindewolf et al., 2023; Züst et al., 2011). Additionally, while not uniform to all NSP16-mutant CoV infection, some studies also detected increases in IFN-I production, which were linked to activation of the cytosolic RNA sensor, melanoma differentiation-associated gene 5 (MDA5) (Russ et al., 2022; Züst et al., 2011). Together, these results indicate that loss of 2′-*O*-MTase activity mediates an IFN-I-based attenuation of CoVs.

Consistent with the data *in vitro*, CoV NSP16 mutants caused less severe disease *in vivo*, varying across different animal models (Menachery et al., 2017, 2014b; Schindewolf et al., 2023; Ye et al., 2022; Züst et al., 2011). For MHV, the NSP16 mutant (NSP16-D130A) was highly attenuated after intraperitoneal injection with no detectable titer in either the liver or spleen (Züst et al., 2011); notably, a mutant lacking capacity to bind cap0 RNA (NSP16-Y15A) was similarly attenuated. Both BALB/c and C57BL/6 mice infected with the SARS-CoV NSP16-D130A mutant also displayed a sharp reduction in viral titer at later timepoints (4- and 7-days post-infection, DPI), reduced weight loss, and less diseased lung function than control mice (Menachery et al., 2014b). Moreover, similar gene expression in the lungs was observed at 1 and 2 DPI; decreases in immunity-related genes were observed in mutant-infected mice at 4 and 7 DPI, consistent with viral clearance (Menachery et al., 2018). For MERS-CoV, the NSP16-D130A mutant was attenuated (Menachery et al., 2017) in two different murine models (Cockrell et al., 2016; Zhao et al., 2014), showing *a* >2 log_10_-lower viral titer in the lung at 4 DPI, compared to wild-type (WT)-infected mice. Finally, SARS-CoV-2 NSP16-D130A mutants were attenuated in both a hamster model (Schindewolf et al., 2023; Ye et al., 2022) and a transgenic human ACE2-expressing mouse model (McCray et al., 2007; Ye et al., 2022). In each model, SARS-CoV-2 2′-*O*-MTase mutants had reduced viral load in the lung at 4 DPI and less lung disease than control animals. While immune responses varied across studies in the hamster model, viral attenuation was observed most strongly in the upper *versus* lower airway (Schindewolf et al., 2023; Ye et al., 2022), suggesting differential immune responses between tissues. Together, studies with NSP16 2′-*O*-MTase mutants demonstrate an essential function to CoVs which is necessary for infection and pathogenesis.

## 6 IFIT proteins restrict CoV NSP16 mutants

Studies of CoV MTase mutants have underscored the importance of IFIT family members, especially IFIT1, in mediating attenuation of NSP16-deficient CoVs (Abbas et al., 2017; Menachery et al., 2017, 2014b; Russ et al., 2022; Schindewolf et al., 2023; Züst et al., 2011). IFIT family members are highly expressed during IFN-I stimulation, and are also induced by interferon regulatory factor (IRF)−3 (Grandvaux et al., 2002); therefore, they are an important component of the early antiviral response. IFIT proteins have different affinities for RNA cap structures (Kumar et al., 2014), which can be modulated by their interactions with each other (Johnson et al., 2018) in a species-dependent manner. Human IFIT1 recognizes cap0 structure (Abbas et al., 2017), *i.e.* an RNA cap lacking 2′-*O*-methylation, and can homodimerize or bind IFIT2 or IFIT3; it also forms a trimer with both IFIT2 and IFIT3 (Mears and Sweeney, 2018).

Because IFIT1 binds cap0 RNA, it competes with eukaryotic initiation factor (eIF) 4F for binding of RNA cap, impeding 48S ribosomal complex formation and thereby inhibiting translation of cap0 RNA (Kumar et al., 2014). Moreover, IFIT1 was also shown to interact with eIF3 via a yeast two-hybrid screen, and exogenous expression of IFIT1 suppressed translation of a reporter construct in a manner dependent on this interaction (Guo et al., 2000). These findings suggest a model of IFIT1 inhibition of cap0 RNA whereby IFIT1, which associates with eIF3 (Terenzi et al., 2006), out-competes neighboring eIF4F for binding to cap0 RNA, thus restricting translation of cap0 RNA. In addition to its sensitivity to viral cap0 RNA, IFIT1 can also target host cap0 RNAs (Williams et al., 2020).

Interestingly, IFIT proteins have been shown to also interact with components of the IFN-I induction pathway. IFIT1 binds stimulator of interferon genes (STING) to modulate its interactions with other IFN-I pathway components (Li et al., 2009). IFIT3 immunoprecipitated with both MAVS and TANK binding kinase 1 (TBK1) and was found to be necessary for robust IFNβ induction (Liu et al., 2011). It remains unclear whether the cap-binding activities of IFIT proteins are necessary for their interactions with components of IFN-I pathways, or *vice versa*. Yet, these results indicate that IFIT proteins directly interact with cap0 viral RNA and may also amplify the IFN-I response leading to more antiviral activity.

While a fuller understanding of their role in innate immunity remains incomplete, IFIT family members have a profound effect on the attenuation phenotypes of CoV NSP16 mutants. IFIT1 knockdown partially or fully restored replication *in vitro* of NSP16 mutants of MHV (Züst et al., 2011), SARS-CoV (Menachery et al., 2014b), MERS-CoV (Menachery et al., 2017), and SARS-CoV-2 (Russ et al., 2022; Schindewolf et al., 2023). Consistently, IFIT1 overexpression *in vitro* attenuated NSP16 mutants of HCoV-229E (Abbas et al., 2017) and SARS-CoV-2 (Schindewolf et al., 2023) compared to their respective WT controls. *In vivo*, NSP16-mutant SARS-CoV infection in IFIT1^−/−^ C57Bl/6 mice restored both weight loss over a 7-day infection course and viral titer in the lung at 4 DPI to WT levels (Menachery et al., 2014b). IFIT2 knockdown *in vitro* partially restored replication to NSP16-mutant SARS-CoV (Menachery et al., 2014b), whereas IFIT2 overexpression *in vitro* more strongly attenuated NSP16-mutant *versus* WT MHV (Daffis et al., 2010). Finally, IFIT3, recently shown to augment the ability of IFIT1 to sense cap0 RNA (Johnson et al., 2018), also mediated, in part, the attenuation of NSP16-mutant SARS-CoV-2 *in vitro* (Schindewolf et al., 2023). Overall, these results suggest that the attenuation phenotypes displayed by the various NSP16 2′-*O*-MTase-mutant CoVs are mediated by cap0-sensing and targeting by IFIT family members.

## 7 The role of MDA5 in NSP16 MTase mutant attenuation

**Fig. 2:**
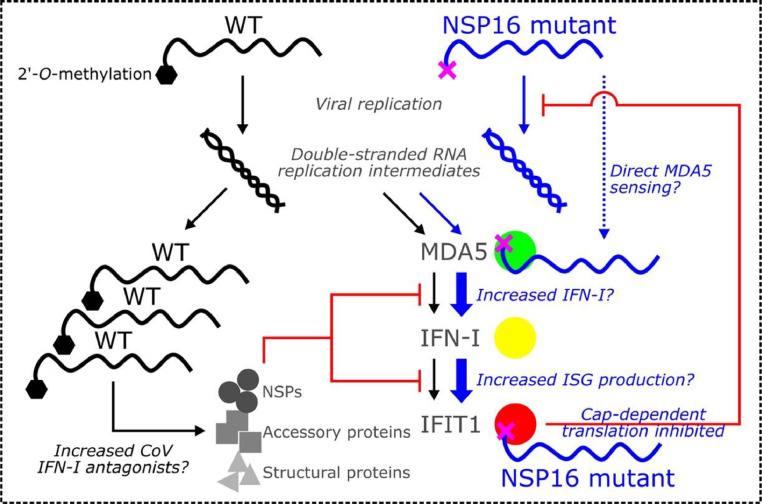
**Model of CoV NSP16 2′-*O*-Methlytransferase Mutant Attenuation.** While CoV double-stranded replication intermediates can be recognized by RIG-I like receptors including MDA5, NSP16-mutant viruses possess RNA lacking 2′-*O*-methylation (purple “X”) on their cap. This RNA lacking 2′O methylation drives attenuation of CoV NSP16 mutants via three, non-mutually exclusive mechanisms: (1) NSP16 mutant RNA is sensed by MDA5 which in turn induces increased type I interferon (IFN)-I production; (2) IFIT1 and other IFIT family proteins directly restricts NSP16 mutant RNA lacking cap 2′-*O*-methylation; (3) restriction of NSP16 mutant RNA reduces production of CoV IFN-I antagonists augmenting the antiviral state. Each of these mechanisms have been linked to NSP16 mutant attenuation and their contribution may vary across cell, tissue, and species type.

Besides IFIT proteins, the cytoplasmic RNA sensor MDA5 also plays a role in recognizing or amplifying the response to CoV RNA cap structures, although the precise mechanism remains unclear. MDA5, a retinoic acid-inducible gene (RIG)-I-like sensor helicase, is a primary sensor of CoVs (Rebendenne et al., 2021; Russ et al., 2022; Sampaio et al., 2021; Yin et al., 2021). It is generally believed to recognize long double stranded RNA (> 2 kb) (Kato et al., 2008), but it has also been implicated in recognizing cap0 RNA which lacks 2′-*O*-methylation (Russ et al., 2022; Züst et al., 2011). MDA5 subsequently interacts with MAVS to initiate pathways leading to IFN-I induction (Kawai et al., 2005). Using an MHV NSP16 mutant, MDA5 was found to mediate attenuation of viral replication resulting in increased IFN-I induction via IRF-3 activation (Züst et al., 2011). However, MDA5 knockout failed to restore NSP16-mutant MHV replication *in vivo*. For SARS-CoV NSP16 mutants, loss of MDA5 had no major effect on restoring replication *in vitro* or *in vivo* (Menachery et al., 2014a, 2014b); yet, MDA5^−/−^ mice had restored lung disease with NSP16-mutant virus infection. Most recently, the increase in IFN-I induction by a SARS-CoV-2 NSP16 mutant was abrogated by nullifying MDA5, although viral replication was not restored to WT levels (Russ et al., 2022). Notably, all CoV NSP16 mutants targeting 2′-*O*-MTase function remain susceptible to IFN-I pre-treatment prior to infection, suggesting that the role of MDA5 in amplifying the innate immune response may be key to its role in antagonizing NSP16-mutant CoVs. Therefore, MDA5 appears to indirectly antagonize NSP16-mutant CoVs, stimulating induction of IFN-I rather than directly exerting antiviral effects. Additionally, WT CoVs, which are less sensitive to IFN-I than NSP16 mutants, may be able to suppress IFN-I induction by virtue of better viral replication and therefore greater production of IFN-I pathway-suppressing viral proteins that exist in the CoV repertoire (Xia et al., 2020) (Fig. 2).

## 8 Therapeutically targeting NSP16 MTase function

Given the importance of 2′-*O*-MTase function and the availability of viral MTase structural data (Byszewska et al., 2014; Klima et al., 2022; Krafcikova et al., 2020; Rosas-Lemus et al., 2020; Viswanathan et al., 2020), CoV NSP16 would appear to be an attractive target for antiviral drug development . With the exception of nirmatrelvir (Paxlovid) targeting the main protease (Mpro) (Hoffman et al., 2020), antiviral drug development for emergent CoVs has typically relied on repurposing drugs designed for other pathogens, such as the nucleoside analogs remdesivir (Sheahan et al., 2017) and molnupiravir (Sheahan et al., 2020).

Using *in silico* approaches with available structural data (Bobrovs et al., 2021), compounds that bind either the SAM-binding site of NSP16 (Sulimov et al., 2022), the RNA cap-binding site, or both in a bi-substrate mechanism of action (Bobileva et al., 2023; Devkota et al., 2021) have been identified as therapeutic candidates; some candidates have also been evaluated for potential cross-reactivity with host MTases (Bobiļeva et al., 2021). Additionally, methods for high-throughput screening of compounds *in vitro* with activity against SARS-CoV-2 NSP16 have been described (Khalili et al., 2021; Samrat et al., 2023). Building from these screening platforms, several studies have identified compounds with demonstrated activity against NSP16 (Bergant et al., 2022; Bobiļeva et al., 2021; Bobrovs et al., 2021; Li et al., 2023a; Omer et al., 2023). Tubercidin, an adenosine analog, has specificity toward NSP16 2′-*O*-MTase activity and reduces SARS-CoV-2 replication (Bergant et al., 2022). However, while tubercidin reduced viral load *in vivo*, drug toxicity led to accelerated weight loss. Sinefungin, a SAM analog, also reduced SARS-CoV-2 replication in a dose-dependent manner that was augmented by IFN-I treatment (Schindewolf et al., 2023). Similarly, chemically modified analogs of SAM have shown effectiveness at inhibiting NSP16 *in vitro* (Bobiļeva et al., 2021). Beyond direct NSP16 inhibition, targeting the SAM cycle may also provide an indirect means to interfere with both viral MTases (NSP14, NSP16) as well as possible host MTases (MTr1) CoVs may rely on (Bergant et al., 2022). 3-deazaneplanocin A (DZNep) inhibits *S*-adenosyl-l-homocysteine (SAH) hydrolase and acts as a competitive inhibitor of MTases (Glazer et al., 1986). Studies have demonstrated inhibition of SARS-CoV-2 by DZNep *in vitro* with some efficacy *in vivo* (Bergant et al., 2022; Kumar et al., 2022). Together, the data indicate that therapeutically targeting NSP16 2′-*O*-MTase activity can be an effective route to treat CoV infection.

While targeting NSP16 has shown some efficacy, combinations with other therapeutics may offer an even more promising approach. Since NSP16-mutant CoV attenuation is dependent on intact IFN-I pathways and production of IFIT family members (Schindewolf et al., 2023; Züst et al., 2011), synergy between NSP16 inhibitors and IFN-I-targeted therapeutics may be particularly promising. For example, DZNep was shown to synergize with IFNα and remdesivir treatment *in vitro* for an enhanced antiviral effect (Bergant et al., 2022). Similarly, an additive antiviral effect was observed upon treatment with sinefungin and IFN-I together (Schindewolf et al., 2023). Importantly, synergy between these treatments may be particularly effective *in vivo*. While antiviral treatments *in vivo* have been shown to reduce titers within the lung (Kuroda et al., 2023; Pruijssers et al., 2020), recent studies have shown NSP16-mutant SARS-CoV-2 to be more attenuated in nasal wash (Schindewolf et al., 2023), where replication of WT virus is particularly robust (Zhou et al., 2021). The results suggest increased IFN-I signaling and interferon-stimulated gene (ISG) responses in the nasal passages could possibly enhance NSP16-targeted therapeutics. If true, development of a nasal spray containing an NSP16 2-*O*-MTase inhibitor could be attractive approach. While additional studies are required, NSP16 2-*O*-MTase activity represents a highly conserved viral function to target for treatment of both current and future CoVs.

## 9 NSP16-Deficiency as a basis for live attenuated vaccines

In addition to presenting an attractive target for antiviral development, NSP16 could also form the basis for a live attenuated vaccine (LAV) platform. NSP16 2′-*O*-MTase mutants of SARS-CoV and MERS-CoV protected mice from lethal challenge with control virus (Menachery et al., 2017, 2014b). In both studies, serum neutralizing antibody titer induced by vaccination correlated with protection. Recently, a SARS-CoV-2 NSP16 mutant protected hamsters challenged with a clinical SARS-CoV-2 isolate; the NSP16-based LAV afforded sterilizing immunity in both the lung and nasal wash (Ye et al., 2022). Despite this success, however, an attenuated vaccine based on NSP16 mutation alone may still pose a risk in susceptible populations. Prior studies with a SARS-CoV NSP16 mutant showed susceptibility of aged mice to the mutant at high doses and reversion to virulence in immunocompromised models (Menachery et al., 2018). Therefore, single targeting of NSP16 as an attenuated CoV vaccine may be problematic.

While single targeting of NSP16 carries significant risks, properties of the NSP16 mutation allow its use in combination with other attenuating mechanisms. Importantly, NSP16 mutants have no major replication attenuation in permissive cell lines such as Vero (Menachery et al., 2017, 2014b; Schindewolf et al., 2023), allowing for the rescue of combination-mutation attenuated strains. One LAV approach for SARS-CoV targeted NSP16 activity in combination with disruption of the CoV exonuclease, NSP14 (Menachery et al., 2018). The double mutant employed multiple attenuating mutations while delivering protection against heterologous challenge, age-dependent disease, and reversion to virulence in immunocompromised models (Menachery et al., 2018). Additionally, recent LAV studies with porcine epidemic diarrhea virus (PEDV) and infectious bronchitis virus (IBV), two important veterinary CoVs, have utilized combination mutations with NSP16 and other viral proteins (Hou et al., 2019; Keep et al., 2022). Several studies have now characterized attenuated mutants of SARS-CoV-2 that could be used in combination with NSP16 for a LAV (Johnson et al., 2021; Liu et al., 2022; Vu et al., 2022). While the success of the mRNA vaccine platform reduces the likelihood of using LAVs in humans going forward, the combination-attenuation platform could have great utility for CoV pathogens in domestic animals, where other LAVs have been successfully deployed (Brun, 2022). In summary, developing LAVs against CoVs that employ inactivation of multiple viral proteins, including NSP16, to both drive attenuation and prevent reversion to virulence, is a promising strategy.

## 10 Exploring other roles of NSP16

Beyond the 2′-*O*-MTase function of NSP16, NSP16 may still have other roles that warrant additional exploration. Supporting this concept, CoV mutants engineered without NSP16 could not be recovered (Almazán et al., 2006; Schindewolf et al., 2023), and no CoV mutants lacking NSP16 have been reported. One explanation is that NSP16 is a key structural component of the CoV RTC. For example, there is preliminary evidence that NSP16 can form a trimer complex with both NSP10 and NSP14, and that this interaction modulates the exonuclease activity of NSP14 (Matsuda et al., 2022). Furthermore, computational modeling of the SARS-CoV-2 RTC suggests that NSP16 may form an integral component of a large complex involving NSP7, NSP8, NSP10, NSP12, NSP13, NSP14, NSP15, and nucleoprotein (Perry et al., 2021). NSP16 may also play a role in host modulation. SARS-CoV-2 NSP16 was shown to bind U1 and U2 small nuclear RNAs (snRNAs) to disrupt splicing of host mRNAs (Banerjee et al., 2020). This interaction is likely mediated by the RNA-binding site of NSP16, but the physical basis for this interaction remains to be explored, as well as whether 2′-*O*-MTase function is dispensable for this interaction. Thus, while it may be essential for CoV 2′-*O-*MTase activity, NSP16 may play other critical roles in viral replication and host translation. Further dissection of these aspects of NSP16 would aid our understanding of CoV infection and possibly lead to additional CoV treatments.

## 11 Conclusion

CoV NSP16, via a conserved catalytic tetrad, provides 2′-*O*-MTase function essential to evasion of host innate immunity. Studies with CoVs harboring a mutation in its catalytic tetrad demonstrated attenuation *in vitro* and *in vivo* primarily due to the activity of IFIT family members, which sense a lack of cap-proximal 2′-*O*-methylation on viral RNA. Antiviral treatments targeting NSP16 function are promising drug candidates and can be used in combinatorial approaches with other therapeutics. Moreover, NSP16 deficiency has shown potential as a part of a live attenuated CoV vaccine platform. In summary, targeting NSP16 disrupts a key aspect of CoV immune evasion and offers a highly conserved enzymatic function that holds promise as a target for mitigating future CoV outbreaks.

## Funding

VDM was supported by grants from NIAID of the NIH (R01-AI153602, R21-AI145400, and U19AI171413) and by a STARs Award provided by the University of Texas System. CS was supported by NIAID of the NIH (T32-AI007526) and by a grant from the Institute of Human Infections and Immunity at UTMB COVID-19 Research Fund.

## CRediT authorship contribution statement

**Craig Schindewolf:** Conceptualization, Formal analysis, Funding acquisition, Investigation, Project administration, Supervision. **Vineet D. Menachery:** Conceptualization, Formal analysis, Funding acquisition, Project administration, Supervision.

## Declaration of Competing Interest

The authors declare the following financial interests/personal relationships which may be considered as potential competing interests: VDM has filed a patent on the reverse genetic system and reporter SARS-CoV-2. Other author declare no competing interests.

## Data Availability

No data was used for the research described in the article.
